# Diiron(I) Bis(cyclopentadienyl) Complexes with Bridging Iminium Ligands: From Foundational Organometallic Chemistry to Unique Reactivity and Biological Potential

**DOI:** 10.1021/acs.accounts.6c00038

**Published:** 2026-03-26

**Authors:** Sara Benetti, Alessia Cinci, Chiara Zappelli, Fabio Marchetti

**Affiliations:** Department of Chemistry and Industrial Chemistry, University of Pisa, Via Giuseppe Moruzzi 13, 56124 Pisa, Italy

## Abstract

Dimetallic complexes offer a remarkable platform to probe metal–metal cooperativity, enabling ligand reactivity patterns that are inaccessible to mononuclear systems. Starting from [Fe_2_Cp_2_(CO)_4_] (Fp_2_, Cp = η^5^-C_5_H_5_), diiron μ-aminocarbyne (iminium) complexes are available through a straightforward multigram-scale procedure. Carbonyl removal is key to enabling selective modification of the ligand set and promoting the formation of uncommon hydrocarbyl ligands involving the carbyne center. In this context, the insertion of terminal alkynes into iron–carbynes bond affords a wide diversity of vinyliminium complexes, characterized by a highly versatile and modular reactivity with reasonably broad reaction scopes. Specifically, three representative transformations are discussed in this Account: 1) Cyanide addition, leading to a cyano-aminoallylidene ligand, in which an intramolecular amine–CO interaction dictates the stereochemical outcome and facilitates subsequent thermal CO dissociation, thereby enabling further reaction pathways. 2) Incorporation of a selenium atom through vinyliminium deprotonation, yielding intrinsically stable complexes bearing an almost pure selenolate function. This moiety displays marked nucleophilic reactivity, including facile dimerization to a Fe_4_ framework via selenide-to-diselenide oxidation, as well as the construction of a selenophene-decorated Fischer alkylidene ligand. Mild hydrolytic cleavage breaks the alkylidene bridge, providing access to a new family of highly functionalized selenophenes. 3) Vinyliminium deprotonation, representing a key entry point to the first family of ferrabenzenes. A multicomponent assembly involving one carbonyl ligand and ethyl diazoacetate generates a six-membered metallacycle, which is ultimately converted into substituted ferrabenzenes through O-alkylation.

Beyond their organometallic reactivity, cationic aminocarbyne and vinyliminium complexes display a combination of properties that are highly attractive for medicinal applications, including straightforward synthesis, air and aqueous stability, broad structural tunability, and amphiphilicity. These features prompted their evaluation as anticancer agents. Their cytotoxicity relies on a molecular “time bomb” behavior, as extensive fragmentation of the diiron scaffold occurs intracellularly, releasing reactive iron­(I) species and carbon monoxide. The resulting fragments primarily induce mitochondrial dysfunction, leading to disruption of cellular redox homeostasis. Importantly, both cytotoxicity and mechanism of action can be regulated by the choice of substituents and ligands, and appreciable cancer cell selectivity is generally achieved. Notably, selected complexes confirmed their promise in 3D cellular models and, in one case, *in vivo*, warranting further development of these diiron-based anticancer agents.

Overall, this Account traces a long-term research journey centered on diiron bis­(cyclopentadienyl) complexes. The narrative begins in a historical context where organometallic chemistry was largely confined to inert-atmosphere manipulation and biological or aqueous applications were scarcely envisioned. It then progresses through the discovery of novel organometallic reactivity patterns and motifs enabled by metal–metal cooperativity, with emphasis on the most recent advances, and culminates in the transition toward biological applications. Collectively, these studies illustrate how fundamental organometallic chemistry can naturally evolve into the concepts and principles of modern bioorganometallic chemistry.

## Graphical abstract

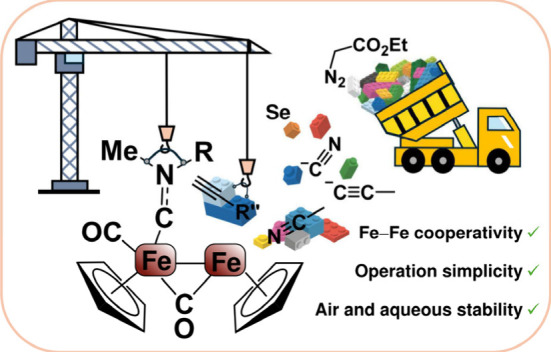

## Key References

- Provinciali, G.; Bortoluzzi, M.; Funaioli, T.; Zacchini, S.; Campanella, B.; Pampaloni, G.; Marchetti, F. Tetrasubstituted Selenophenes from the Stepwise Assembly of Molecular Fragments on a Diiron Frame and Final Cleavage of a Bridging Alkylidene. *Inorg. Chem*. **2020**, 59, 17497–17508.^95^ This work reports the synthesis of highly functionalized selenophenes via a stepwise assembly of small molecular fragments on a diiron scaffold. The process culminates in a coordinated selenophene–alkylidene ligand, which can be cleanly released under mild hydrolytic conditions.
- Biancalana, L.; De Franco, M.; Ciancaleoni, G.; Zacchini, S.; Pampaloni, G.; Gandin, V.; Marchetti, F. Easily Available, Amphiphilic Diiron Cyclopentadienyl Complexes Exhibit in Vitro Anticancer Activity in 2D and 3D Human Cancer Cells through Redox Modulation Triggered by CO Release. *Chem. Eur. J*. **2021**, 27, 10169–10185.^24^ This article describes the synthesis of amphiphilic diiron aminocarbyne complexes, their physicochemical behavior in aqueous media, and their anticancer activity in 2D and 3D cellular models. While only moderate activity was observed in 2D cultures, the complexes displayed marked selectivity toward cancer cells and retained their efficacy in 3D models, reaching, and in some cases surpassing, cisplatin’s performance.
- De Franco, M.; Biancalana, L.; Zappelli, C.; Zacchini, S.; Gandin, V.; Marchetti, F. 1,3,5-Triaza-7-phospha­adamantane and Cyclohexyl Groups Impart to Di-Iron­(I) Complex Aqueous Solubility and Stability, and Prominent Anticancer Activity in Cellular and Animal Models. *J. Med. Chem*. **2024**, 67, 11138–11151.^58^ This study presents the rational design of water-soluble and stable diiron aminocarbyne complexes with enhanced anticancer properties. The lead compound FEACYP combines excellent aqueous solubility and stability with potent and selective cytotoxicity and, *in vivo*, suppresses tumor growth comparably to cisplatin while exhibiting reduced systemic toxicity.
- Benetti, S.; Nottoli, T.; Xiao, Z.; Funaioli, T.; Zacchini, S.; Gasser, G.; Lipparini, F.; Marchetti, F. Multicomponent Synthesis on a Diiron Platform of Stable Ferrabenzenes with Promising Anticancer Activity. *Angew. Chem., Int. Ed*. **2025**, 64, e202510795.^112^ This work reports the first straightforward multicomponent synthesis of stable ferrabenzenes enabled by a cooperative diiron platform. The resulting complexes are air- and water-stable and display promising *in vitro* anticancer activity associated with perturbation of cellular redox homeostasis.

## 1 Introduction

Diiron carbonyl complexes occupy a prominent position at the interface between organometallic chemistry and bioinorganic catalysis. In particular, the discovery of the structure of the [FeFe]-hydrogenases^1^ in the late 1990s has inspired the synthesis and exploration of a wide range of structural and functional mimics of the enzymatic active site, aimed at developing efficient electrocatalysts for H_2_ production. Studies have focused especially on [Fe_2_(μ-dithiolate)]­carbonyl systems, which have been extensively reviewed elsewhere.^2–4^

Well before the rise of bioinspired diiron chemistry, the readily available diiron precursor [Fe_2_Cp_2_(CO)_4_] (Fp_2_, Cp = η^5^-C_5_H_5_) had already established a central role in organometallic chemistry. First reported in 1955,^5^ it emerged as a versatile starting material, affording a wide variety of diiron and monoiron derivatives.^6–10^

In this context, building on the idea that a dimetallic core can serve as a simplified molecular model for a metallic surface, diiron Fp_2_ derivatives bearing bridging hydrocarbyl ligands were employed to elucidate key mechanistic aspects of the Fischer–Tropsch synthesis (FTS).^11,12^ FTS is a key industrial process primarily catalyzed by iron and cobalt, involving the reductive polymerization of carbon monoxide with H_2_ to form hydrocarbons via consecutive C–C coupling events.^13,14^ Casey and collaborators explored the reactivity of bridging carbyne complexes in the 1980s (Scheme 1B, structure **1**).^15,16^ Almost contemporaneously, Angelici and Howell and Manning introduced variants with bridging thiocarbyne (structure **2**)^17^ and aminocarbyne (structure **3**)^18,19^ ligands, opening avenues to expand the chemical space of diiron bis­(cyclopentadienyl) systems.^16,20^ In line with the formal treatment of carbyne as a monocationic ligand,^20^ the iron centers in **1**–**3** preserve the +I oxidation state.

**Scheme 1:**
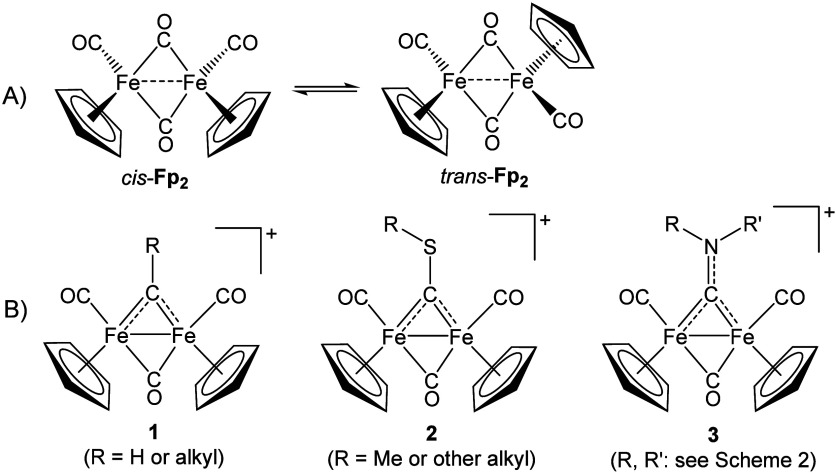
Structures of Diiron Bis­(cyclopentadienyl) Complexes: A) **Fp**_**2**_, Existing in Organic Solvents as Interconverting Cis and Trans Isomers, Defined by the Mutual Arrangement of the Cp Ligands Relative to the Fe_2_(μ-C)_2_ Plane, and B) Cationic Derivatives with a Bridging Carbyne (**1**), Thiocarbyne (**2**), and Aminocarbyne Ligand (**3**, the Focus of This Account)

Due to its strong π-acceptor character, exceeding that of CO, the carbyne moiety occupies a fixed bridging position to maximize backdonation from the iron centers.^21^ This arrangement suppresses the fluxional behavior in solution otherwise typical of Fp_2_ (Scheme 1A).^22^ The backdonation in **3** is relatively weakened by competition of the nitrogen lone pair for the carbyne empty *p* orbital, imparting significant iminium-like character to the CNR­(R′)^+^ ligand (Figure 1).^20^

**Figure 1:**
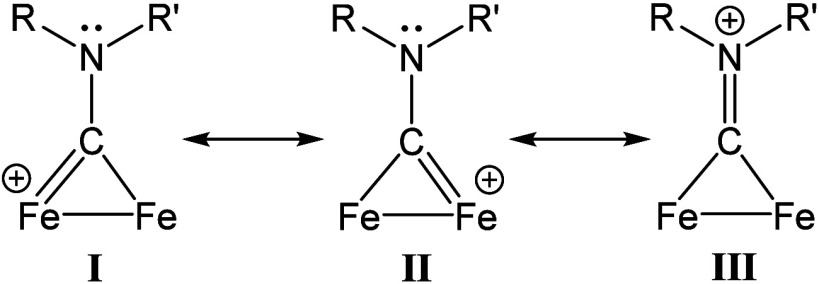
Resonance forms illustrating the hybrid aminocarbyne (I, II)–iminium (III) character of the bridging ligand in complexes **3**.

This is evidenced by both crystallographic data, showing C–N bond distances of 1.28–1.30 Å, and the absence of nucleophilic reactivity of the aminocarbyne nitrogen even in neutral derivatives of **3** (*vide infra*). Conversely, the much less investigated phosphinocarbyne analogue of **3** displays nucleophilicity, enabling phosphorus coordination to other metal centers.^23^ DFT calculations on a selected complex of type **3** (R = Me, R′ = cyclohexyl) confirmed the hybrid aminocarbyne–iminium nature, with a C–N bond order of 1.58.^24^ This peculiar electronic duality underpins the distinctive reactivity patterns discussed throughout this Account.

Over the past 25 years, the exploration of novel reaction pathways in complexes of type **3** and their derivatives has been further stimulated by emerging concepts, such as the growing emphasis on sustainable iron-based transformations^25^ and the recognition of metal–metal cooperativity,^26–30^ giving rise to a rich and multifaceted chemistry that has been partly reviewed elsewhere.^20,31,32^

This Account provides a unifying vision of nearly 50 years of progress, highlighting key conceptual advances and recent biological applications.

## 2 Readily Accessible Diiron Aminocarbyne Complexes

### 2.1 Historical View and General Considerations

Type **3** complexes (<10 combinations of alkyl R/R′ groups) were originally prepared via a two-step synthesis from Fp_2_. CO/isocyanide substitution in benzene or THF yielded [Fe_2_Cp_2_(CO)_3_(CNR)] intermediates,^33^ which were isolated and treated with alkyl triflates or halides to give the final products, recovered by precipitation from the reaction medium or by crystallization.^34,18^

The chemistry of diiron bis­(cyclopentadienyl) complexes was introduced in Bologna by Busetto, a former fellow of Angelici at Iowa State University. Early studies focused on **2** and subsequently turned to **3**.^35–37^ A major impetus came from key improvements implemented in 2001 to the synthetic procedure: although published only in 2018,^38^ the upgraded method has enabled the multigram-scale synthesis of **3** (Scheme 2).^24,39–41^ In this approach, acetonitrile is employed as the solvent for the CO/CNR substitution, performed at room temperature or under reflux depending on the nature of R. Double substitution, favored in the case of aryl isocyanides, is problematic because [Fe_2_Cp_2_(CO)_2_(CNR)_2_] can be alkylated in the second step to produce [Fe_2_Cp_2_(CO)_2_(CNR)­(μ-CNRR′)]^+^ species that are difficult to separate from **3**. To prevent this, Fp_2_ is used in excess (typically 60%), thereby allowing extension of the synthesis to R = aryl. The resulting monoisocyanide complexes, usually obtained as mixtures of interconverting isomers,^33^ are not isolated from unreacted Fp_2_, which does not undergo alkylation. Subsequent alkylation of the [Fe_2_Cp_2_(CO)_3_(CNR)]/Fp_2_ mixture, commonly performed with methyl triflate in dichloromethane, affords a solution that is directly loaded onto an alumina column, allowing recovery of the pure products after elution of Fp_2_ and other impurities. This protocol displays a broad scope, avoids contamination by traces of hazardous isocyanides and methyl triflate, and has been scaled in our laboratory up to 15–20 g of triflate salts in a single batch. Further scale-up appears feasible using appropriate equipment. All complexes **3** are readily obtained as powdery or crystalline solids and can be stored in air in vials for years without detectable degradation.

**Scheme 2:**
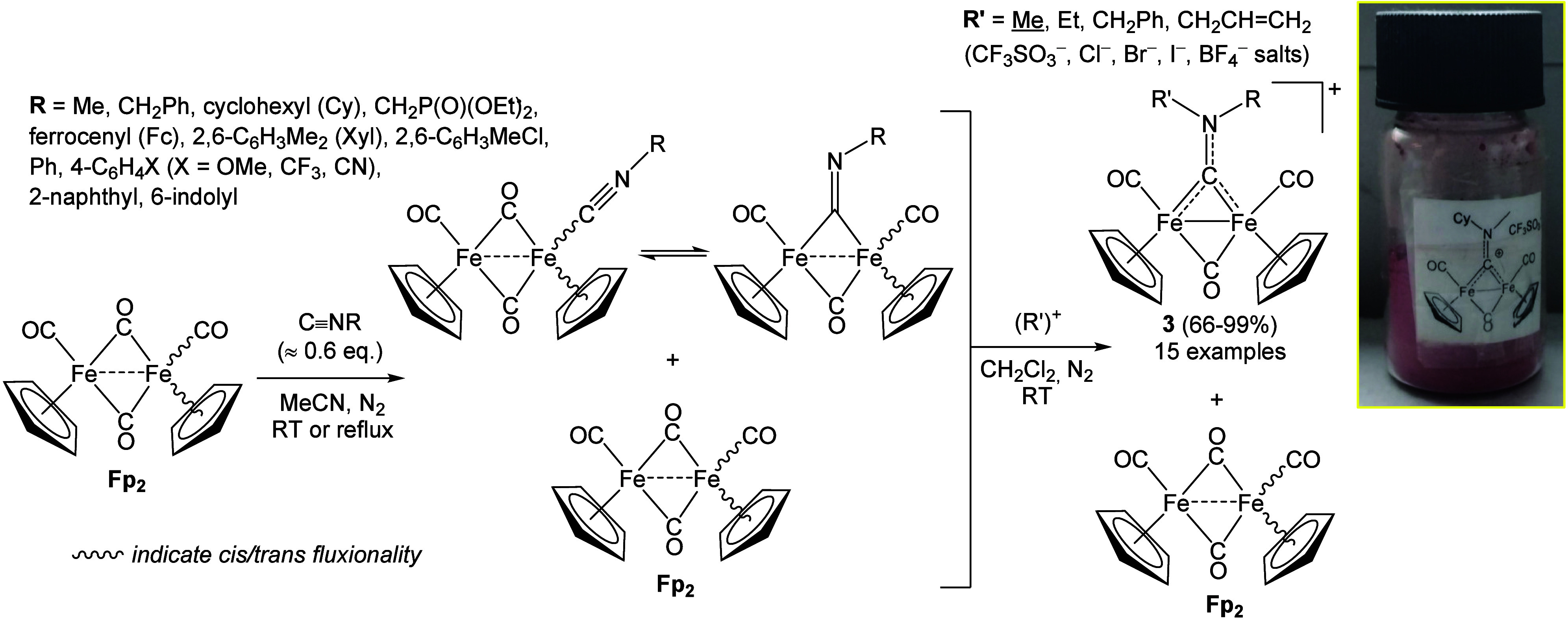
Multigram-Scale Synthesis of Air-Stable Diiron Aminocarbyne Complexes (**3**) via Mono-isocyanide Intermediates (Isolation Unnecessary)^a^

a The inset photograph shows a powdery sample of [Fe_2_Cp_2_(CO)_3_{μ-CNMe­(Cy)}]­[CF_3_SO_3_] (∼4.5 g).

Compounds **3** represent an exceptional class of dimetallic species with a bridging aminocarbyne easily accessible from cost-effective commercial precursors. Notably, their mononuclear counterparts are still unknown: although monoiron-Cp complexes with isocyanide ligands are available, isocyanide alkylation is not observed even when using strong alkylating agents.^42^

Unless otherwise indicated, the cationic diiron complexes discussed hereafter in this Account are triflate salts, with R′ fixed as Me. As a general feature, the chemistry described in the following sections can be conveniently monitored by liquid IR spectroscopy, CO and iminium absorptions being highly sensitive to local structural variations.

### 2.2 CO Substitution and Nitrile Activation

The type **1** complex [Fe_2_Cp_2_(CO)_3_(μ-CH)]^+^ undergoes carbyne coupling with various nucleophiles,^43–45^ whereas the aminocarbyne ligand in **3** is much less reactive due to partial iminium character.^46^ Nucleophilic additions to the aminocarbyne, converting into aminocarbene, are limited to reactions of NaBH_4_ and ^t^Bu_4_NCN with complexes **3** lacking bulky amino substituents.^38,47,48^

Earlier studies indicate that replacing one CO with a labile acetonitrile in M_2_Cp_2_(CO)_3_-based compounds (M = Fe, Ru) markedly affects the reactivity of the dimetallic scaffold.^49^ Building on this, in 2000 Busetto and Zanotti reported a family of nitrile derivatives,^50^ later expanded.^51^ The synthesis is straightforward, exploiting the cationic character of **3**, which weakens Fe→CO π-backdonation and leaves sufficient positive charge on the CO ligands. Accordingly, when an acetonitrile solution of **3** is treated with Me_3_NO,^52^ the latter acts as *O*-nucleophile toward CO; CO_2_ and Me_3_N are generated and vented from the reaction vessel. The resulting metal vacancy is occupied by an acetonitrile molecule (**4**, Scheme 3), and no further CO is removed, even in the presence of a 2-fold excess of Me_3_NO.

**Scheme 3:**
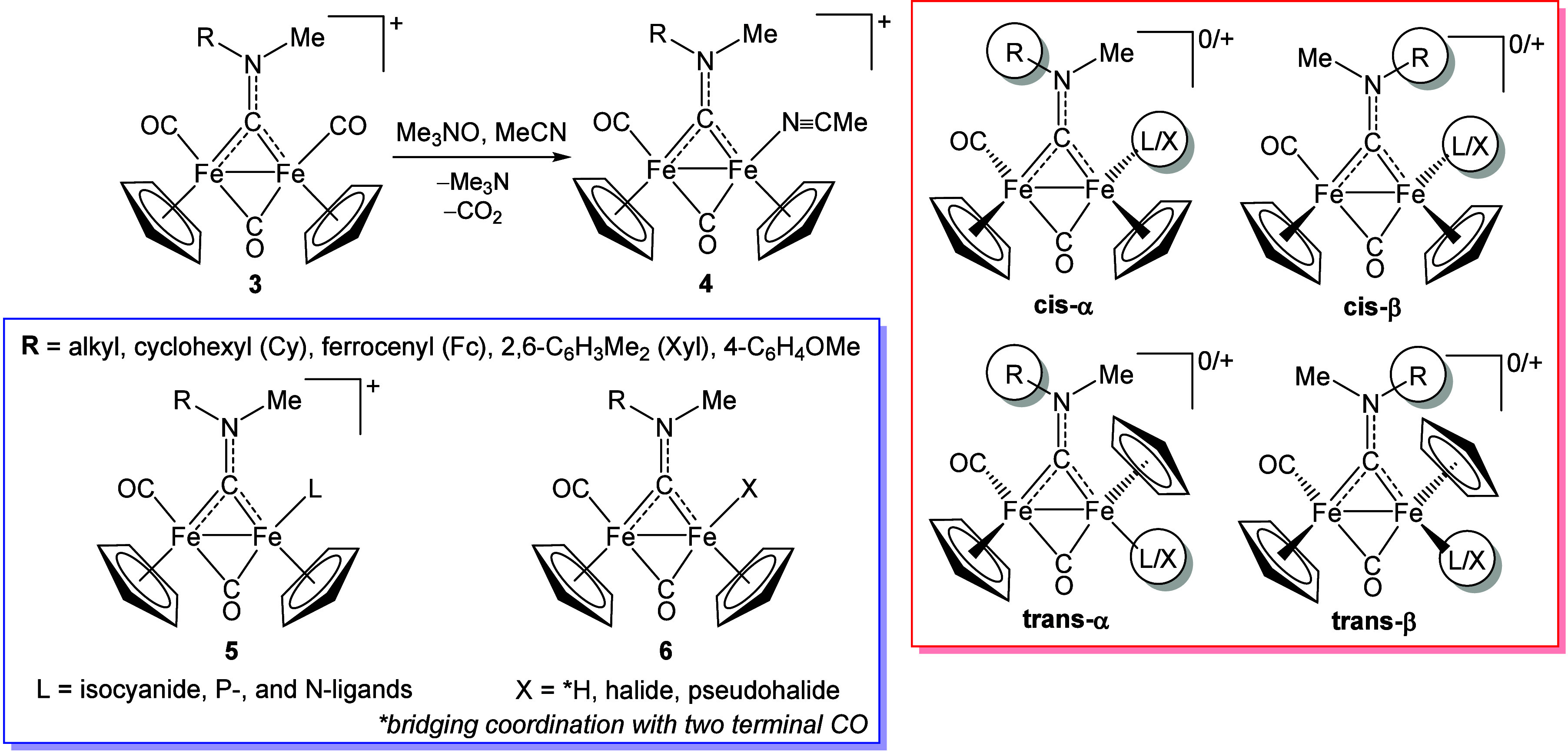
Synthesis of Acetonitrile Complexes **4** Using Trimethylamine-*N*-Oxide^a^

a The blue inset shows cationic and neutral derivatives of **4**. The red inset shows α/β and cis/trans isomerism in complexes **4–6**.

A range of dicarbonyl derivatives of **3**, both cationic (**5**) and neutral (**6**), have been obtained from freshly synthesized complexes **4** via *in situ* acetonitrile substitution with isocyanides,^53,54^ amines,^39,55^ pyridines,^56^ phosphines,^57,58^ hydride,^50^ halides,^24,50^ and pseudohalides^50,59^ (Scheme 3). Some complexes **5**–**6** have been accessed directly from **3** at ca. 60–80 °C.

Complexes **4–6** lose symmetry with nonidentical N-substituents (R ≠ Me), leading to α/β isomerism arising from hindered rotation around the carbyne–nitrogen partial double bond. The bulky R = Xyl minimizes, and in some cases suppresses, the β form. Additionally, cis/trans isomerism, relative to the orientation of the Cp ligands, is observed in some cases, with the trans isomer being particularly relevant for certain neutral compounds **6**.^20,31,59,47^ In complexes **3–6**, the aminocarbyne carbon gives a diagnostic ^13^C NMR resonance at 300–340 ppm.^20^

During exploration of the chemistry of nitrile complexes, we unexpectedly found that alkylnitrile ligands are not displaced upon reaction with strongly basic reagents such as Grignards and lithium alkyls or acetylides (Scheme 4A). Instead, unusual deprotonation^60,61^ occurs to give cyanoalkyl ligands:^62^ the resulting complexes (**7**) are rare examples of Fe_2_Cp_2_ alkyl species stabilized by the electron-withdrawing cyano moiety.^38^ The reaction of *p*-tolylacetylide with **4b**, lacking potentially acidic hydrogens, afforded a distinctive seven-membered diferracycle (**8**) via nucleophilic attack at the nitrile followed by N–C coupling with the carbyne (Scheme 4B).^63^

**Scheme 4:**
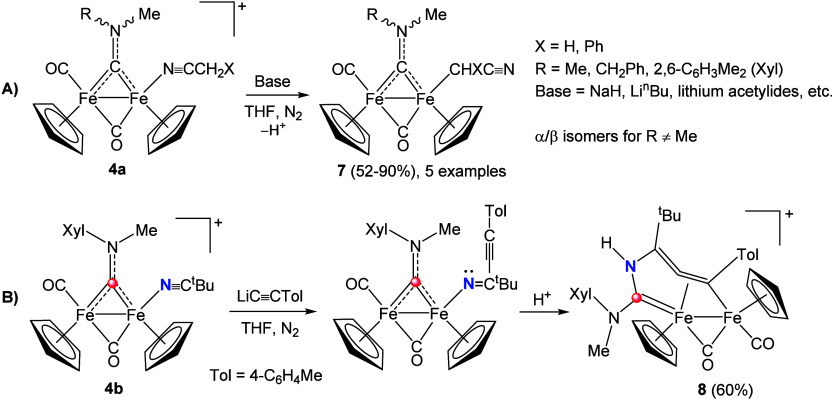
A) Nitrile-to-Cyanoalkyl Deprotonation and B) Acetylide-Promoted Aminocarbyne–Nitrile Coupling

### 2.3 Aminocarbyne Ligand as Hydride Transferor in Catalytic Hydrogenation Reactions

Complexes **3** are coordinatively and electronically saturated (36 electrons), and their robustness is enhanced by tightly bound ligands with complementary electronic properties (CO and aminocarbyne as π-acceptors, Cp as π-donors). This picture disfavors direct involvement of the iron centers in conventional metal-centered catalytic processes. On the other hand, the presence of unsaturated ligands enables ligand-centered catalytic pathways. Accordingly, the cyanide complex **6a** exhibits moderate activity (maximum TOF = 22 h^–1^) in the transfer hydrogenation of a range of ketones and aldehydes using isopropanol as hydrogen donor in the presence of KOH (Scheme 5A).^64^ Spectroscopic and DFT studies highlighted a key role for the aminocarbyne, behaving as a hydride transfer agent from the isopropoxide anion to the carbonyl substrate via transient formation of an aminocarbene intermediate.

**Scheme 5:**
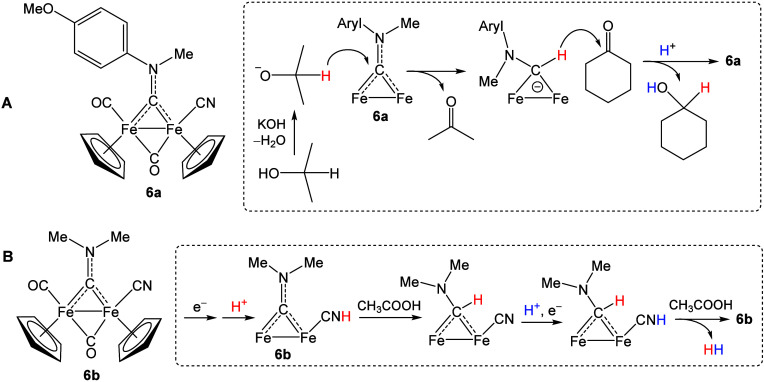
Diiron Aminocarbyne–Cyanide Complexes Investigated in Homogeneous Catalysis and Related Key Steps: A) Transfer Hydrogenation of Ketones/Aldehydes Using Isopropanol and B) Electrocatalytic H_2_ Production from Acetic Acid in Acetonitrile

Complex **6b** has been proposed as a [FeFe]-hydrogenase mimic, catalyzing electrochemical H_2_ production from acetic acid in acetonitrile.^65^ The DFT-derived catalytic cycle involves both carbyne and cyanide as sites for hydrogen addition, with CH_3_COOH acting as a proton shuttle (Scheme 5B).^66^

## 3 Vinyliminium Complexes

### 3.1 Synthesis and General Considerations

In 2001, un unexpected product (**9a**) was isolated from the reaction of **4b** with LiC≡CSiMe_3_ (Scheme 4): the ^13^C NMR spectrum showed disappearance of the downfield carbyne resonance and absence of the NC^t^Bu moiety. Subsequent crystallographic characterization (Figure 2) revealed a bridging C_3_ ligand (vinyliminium), formed via insertion of HC≡CSiMe_3_ into the Fe-carbyne bond.^67,68^

**Figure 2:**
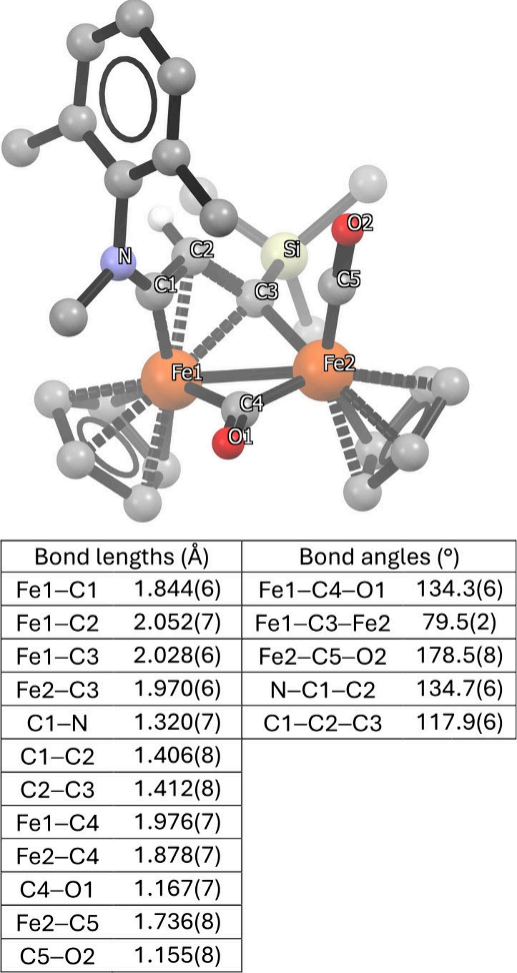
Molecular structure of **9a** (H atoms omitted for clarity, except C^2^H).

The observed alkyne insertion reaction was soon shown to display an almost universal scope for terminal alkynes, enabling the development of complexes **9** as a potentially unlimited family of readily accessible organometallics (Scheme 6A). Since 2003, nearly 150 complexes of this type have been prepared directly via alkyne insertion,^69^ typically on 100 mg–​2 g scales and in good to excellent yields.^20,70–80^

**Scheme 6:**
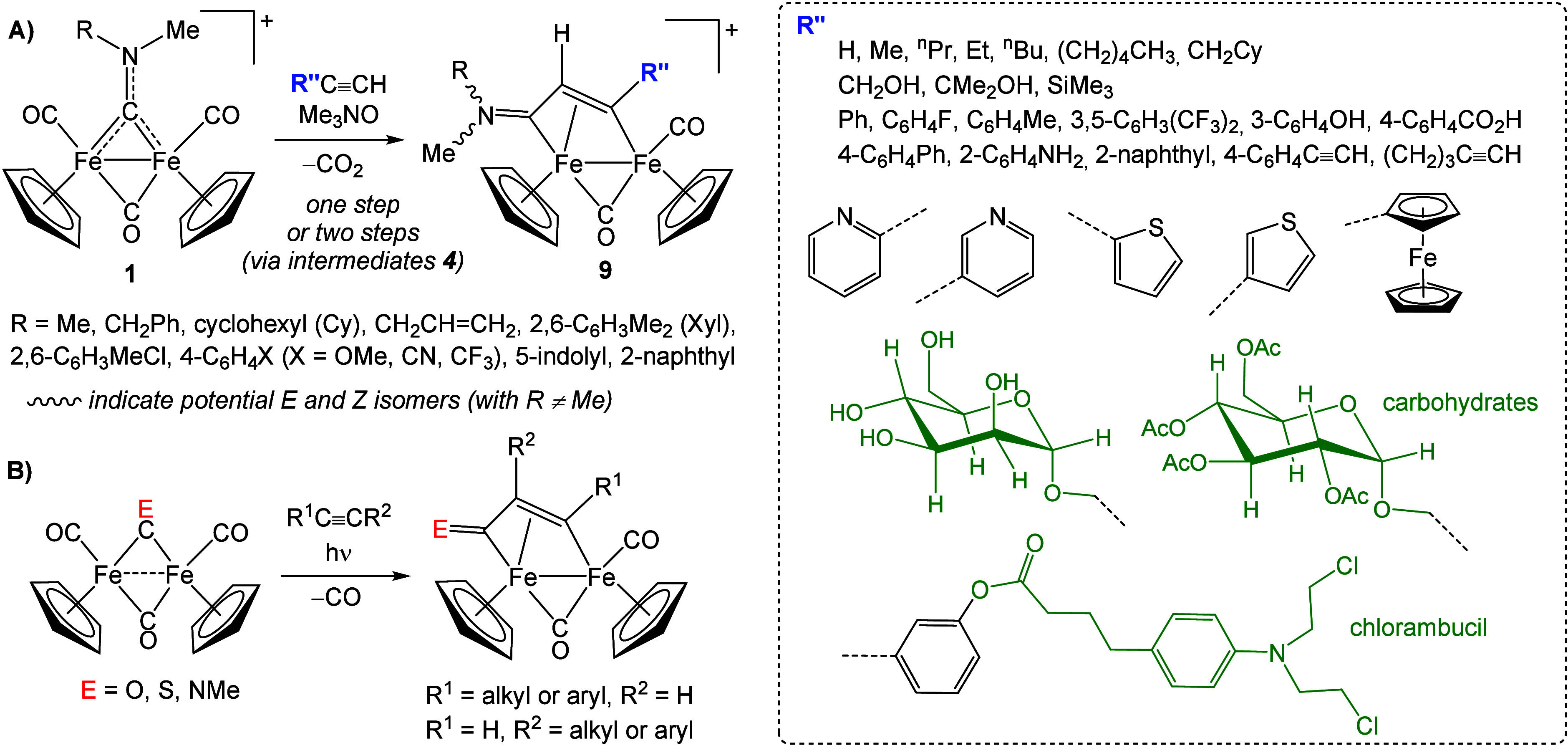
A) Synthesis of Vinyliminium Complexes via Chemical CO Extrusion and Aminocarbyne–Alkyne Coupling^a^ and B) Non-regioselective Alkyne Insertion Reactions via Photolytic CO Elimination

a The inset shows the scope of alkyne substituents, with selected bioactive fragments^78,79^ highlighted in green.

Consistent with the isolobal analogy^81^ between {CNR­(Me)^+^} and {CE} (E = O,^82^ S,^83^ NR^84^) fragments, parallel alkyne insertion processes are also feasible (Scheme 6B). Nevertheless, the synthesis of **9** benefits from three key features:

1) *Regioselectivity*, with R″ positioned away from the iminium group; when R ≠ Me, E/Z stereoisomers may be observed, the E isomer (R oriented away from the Fe_2_Cp_2_ core) being sterically favored.
2) *Cationic nature* of **3**, enabling selective Me_3_NO-mediated CO removal (see Section 2.2) and avoiding the less selective photolytic conditions required for neutral [Fe_2_Cp_2_(CO)_3_(μ-CE)] analogues. Most commonly, complexes **9** are synthesized at room temperature via initial formation of acetonitrile adducts **4**, followed by NCMe-alkyne replacement.
3) *Operational simplicity*, as products are easily purified by alumina chromatography and can be stored in air in vials for years without degradation. The synthetic procedure from **3** to **9**, originally carried out under an inert atmosphere,^67^ was later optimized to be conducted in air.^70^

This extensive structural variability allows the use of tailored vinyliminium complexes as organometallic ligands. Recently, a 3-pyridyl substituent has been exploited to obtain stable diiron–iridium conjugates of biological relevance (Scheme 7).^85^

**Scheme 7:**
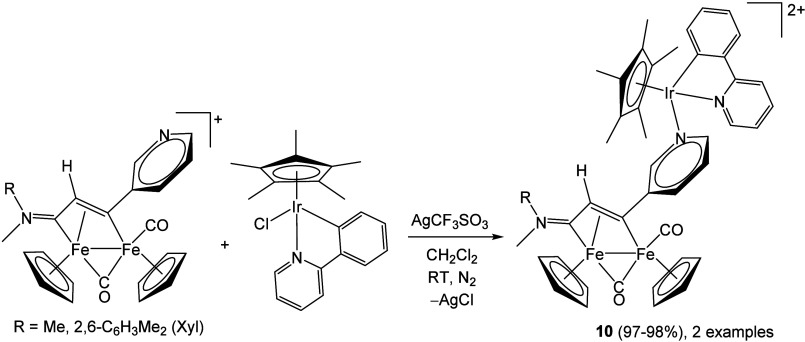
Synthesis of Diiron–Iridium Complexes via a Pyridyl–Vinyliminium Ligand

The vinyliminium ligand in **9** exhibits multisite coordination and extensive charge delocalization, resulting in a rich and finely tunable reactivity (Scheme 8).^20,31^ In general, transformations occur at room temperature, are regioselective, and preserve the C^1^–C^2^–C^3^ connectivity. Cyclization reactions typically involve only one iron center. A notable exception is the formation of functionalized ferrocenes **11–12**,^86,87^ which proceeds with limited regioselectivity via thermal [3+2] coupling of the vinyliminium chain with propargyl alcohols (Scheme 9).

**Scheme 8:**
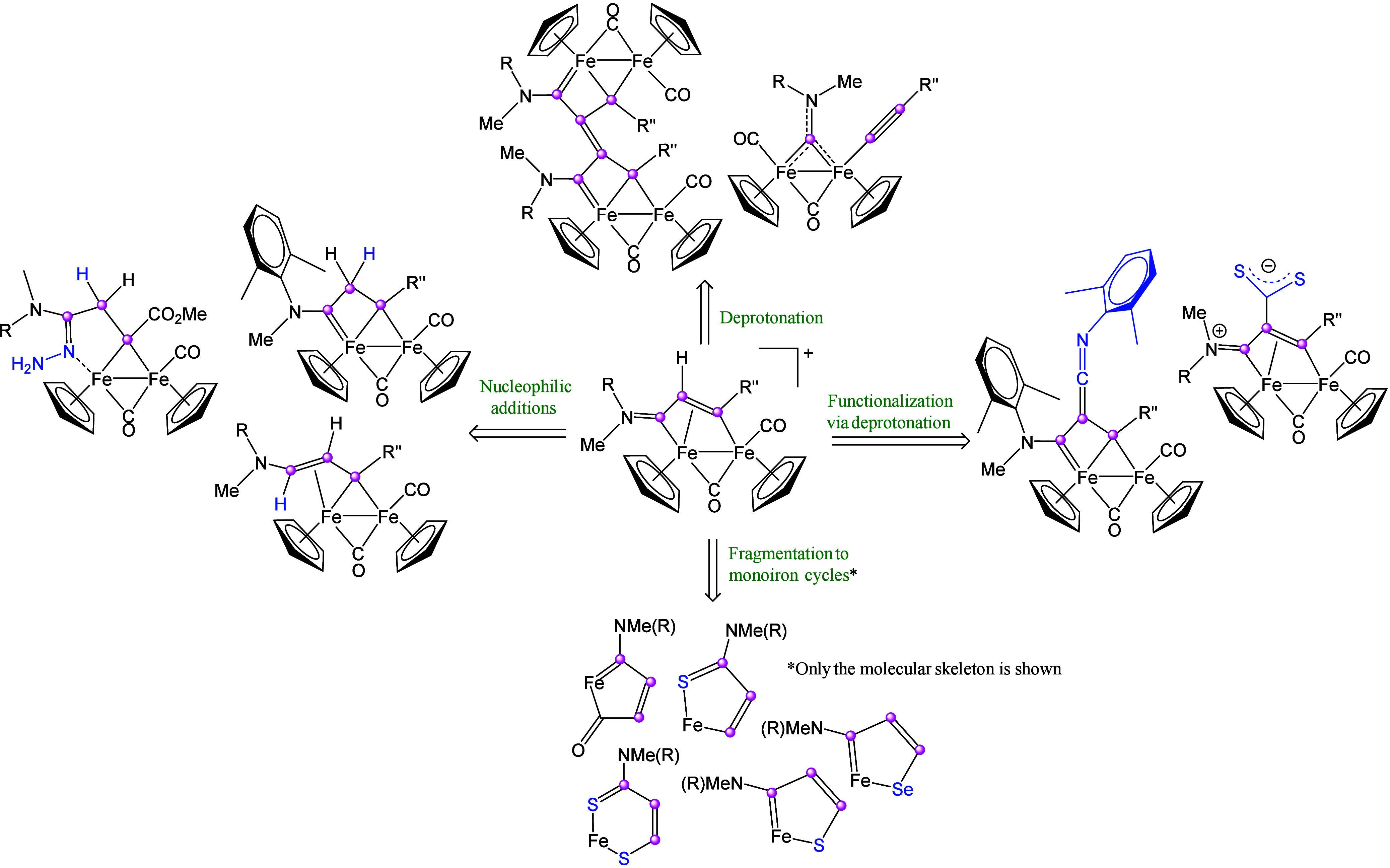
Overview of Main Reactivity Patterns of Vinyliminium Complexes^a^

a Pink dots indicate the original vinyliminium carbons. Reactions shown in Schemes 9–13 are not included.

**Scheme 9:**
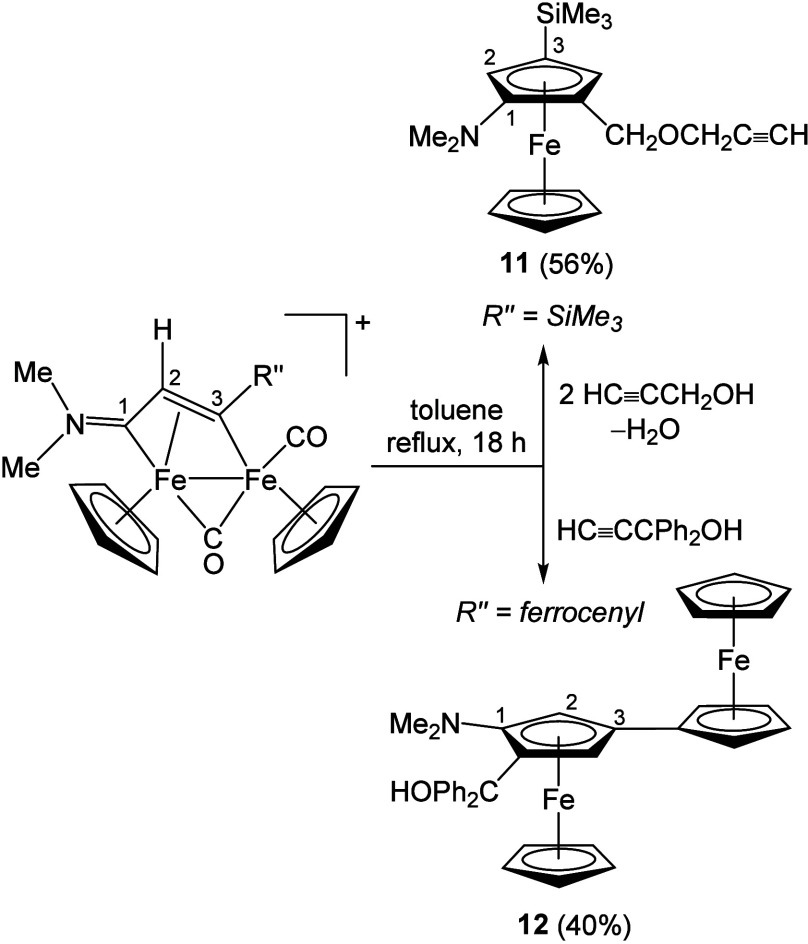
Thermal Coupling of Vinyliminium Ligands with Propargyl Alcohols, Affording Ferrocenes^86^ and Diferrocenes^87^

Selected recent reaction types are discussed below to illustrate key concepts.

### 3.2 Unique Properties Imparted by Cyanide Addition

The air-stable allylidene complexes **13** are synthesized from **9** via cyanide addition to the iminium carbon (R ≠ Xyl), Scheme 10a.^40,88,73,75,89^ Although several dimetallic complexes featuring bridging allylidene ligands have been reported, these typically contain nonfunctionalized C_3_ hydrocarbyl chains.^90,91^ In contrast, the ligand in **13**, bearing both amino and cyano substituents at C^1^, displays distinctive stereochemical features and reactivity. Gas-phase DFT calculations on **13a** (Scheme 10, inset) revealed an intramolecular Lewis acid–base interaction between the terminal CO ligand and the amine nitrogen (N1···C5 distance 2.425 Å), resulting in a significant deviation of the Fe2–C5–O2 angle from linearity (158.37°). For comparison, the corresponding angle in **9a** is 178.5°, Figure 2. This computed interaction is less pronounced in the solid-state structures of **13a** [N1···C5 2.793(2) Å and Fe2–C5–O2 169.9(2)°] and related compounds, likely due to packing effects. Nevertheless, consistent with DFT predictions, the amine–CO contact becomes evident in solution, as indicated by NMR data. This feature imposes one of the two spatial arrangements of the CN and NMe­(R) substituents, rendering the formation of complexes **13** fully stereoselective. Moreover, hindered rotation of the NMe­(R) group around the single C^1^–N bond leads to distinct ^1^H and ^13^C NMR signals for the NMe_2_ unit when R = Me, whereas complexes with R ≠ Me are typically observed as two diastereoisomers.

**Scheme 10:**
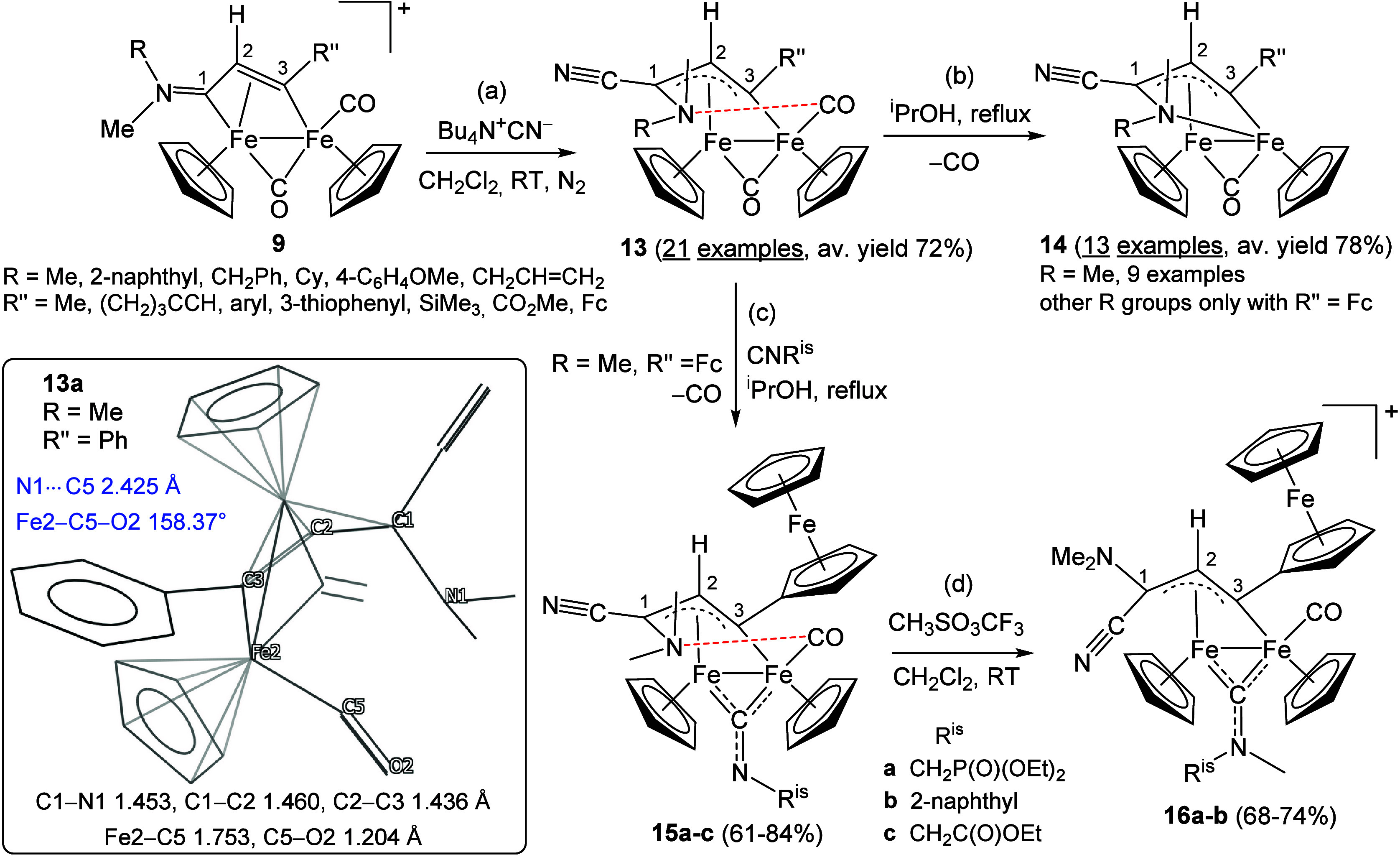
Synthesis and Reactivity of Cyano–Aminoallylidene Complexes^a^

a The inset shows a sketch of the DFT-calculated structure of **13a**.

The partial acyl character acquired by the terminal CO ligand (Figure 3, structure III) weakens the Fe–CO bond, enabling thermal dissociation. In our experience, and in agreement with the literature, CO dissociation from *neutral* diiron bis­(cyclopentadienyl) *dicarbonyl* complexes is challenging due to strong π-backdonation to both Fe–CO bonds, unless harsh conditions promoting extensive decomposition are employed (e.g., aqueous media). Notably, clean CO substitution via either thermal treatment or Me_3_NO strategy is not feasible even for the *cationic dicarbonyl* complexes **9**.

**Figure 3:**
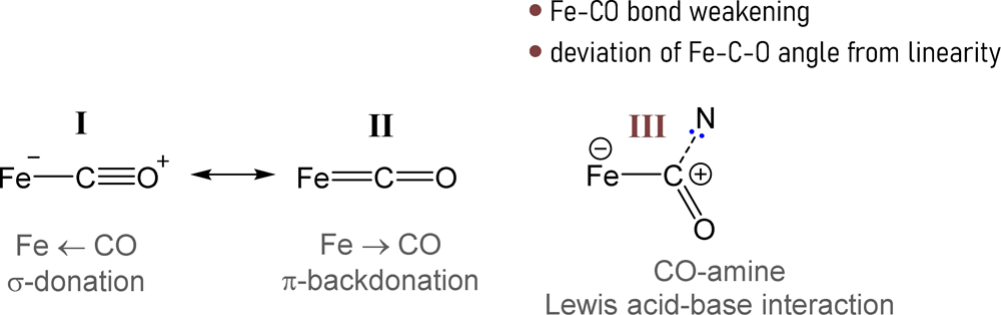
Qualitative representations of the bonding of the terminal CO ligand in complexes **13**. Structures I–II depict canonical Fe–CO resonance descriptions, whereas structure III is a qualitative polarization model illustrating the partial acyl character arising from intramolecular N→CO interaction.

Heating isopropanol solutions of complexes **13** yields the monocarbonyl derivatives **14**, in which the tertiary amine substitutes the dissociating CO ligand (Scheme 10b).^92^ The resulting multisite ligand exhibits weak hemilabile behavior: partial amine displacement was detected by IR spectroscopy in the presence of stoichiometric amounts of PhSSPh or PTA under refluxing isopropanol. Given the established relevance of ligand hemilability in catalysis,^93^ the catalytic potential of complexes **14** was explored in several organic transformations. Selected members of the series catalyze epoxide/CO_2_ coupling at ambient temperature and CO_2_ pressure.^92^ In addition, some type **14** complexes efficiently catalyze the borylation of benzaldehyde with pinacolborane at room temperature.^92^ This reactivity was extended to a range of aldehydes and ketones using the most active catalyst, **14a**, affording the corresponding borate esters. The proposed mechanism involves coordination of the carbonyl substrate to iron, followed by borane addition, presumably assisted by intramolecular amine–carbonyl interaction.

High-temperature reactions of ferrocenyl-decorated complexes **13** with isocyanides afford CO-substitution products, **15a**–**c** (Scheme 10c).^94^ Following dissociation of the terminal CO, the remaining carbonyl migrates to a terminal position, enabling interaction with the amine moiety analogous to that observed in **13**. Therefore, the NMe_2_ group resonates as two distinct signals in the NMR spectra (e.g., 2.27 and 1.75 ppm in the ^1^H NMR spectrum of **15a** in CDCl_3_), indicative of inhibited rotation around C1–N. The incoming isocyanide then adopts a bridging coordination mode, favoring subsequent alkylation. Indeed, reaction with methyl triflate generates a new aminocarbyne ligand (complexes **16a**–**b**, Scheme 10d). This conversion implicates a substantial rearrangement of the diiron framework, accompanied by exchange of the CN and NMe_2_ orientations. As a result, free rotation of the NMe_2_ group is restored, appearing as a singlet in the NMR spectra (2.55 ppm in the ^1^H NMR spectrum of **16a**). The X-ray structures of **15a** and **16a** are compared in Figure 4, highlighting the substantial elongation of the C6–N3 bond upon conversion from isocyanide to aminocarbyne character.

**Figure 4:**
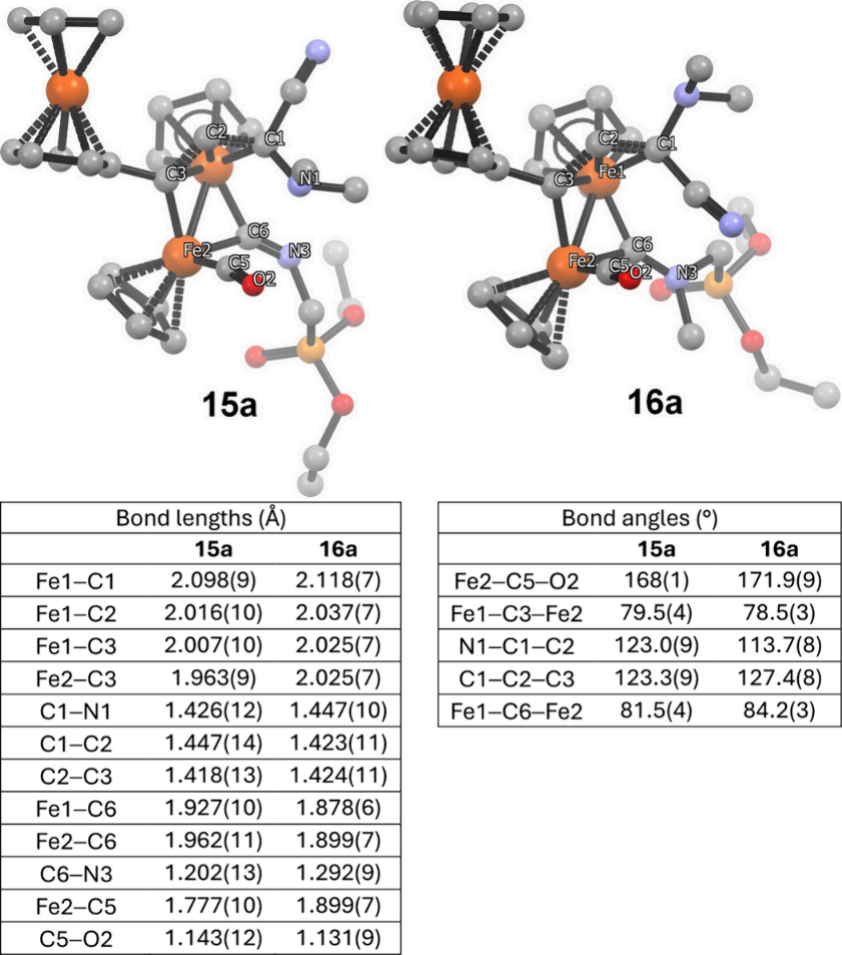
Comparison of the molecular structures of **15a** and **16a** (H atoms omitted).

### 3.3 Selenium Incorporation and Synthesis of Functionalized Selenophenes

Complexes **9** display acidic C^2^-hydrogen and can be deprotonated using medium-to-strong bases. Performing this reaction in a THF suspension of gray selenium affords the zwitterionic complexes **17** in good yields (Scheme 11a).^41,95–97^ As a steric consequence of H^+^/Se exchange, the predominant orientation of the iminium substituents inverts when R ≠ Me. Compounds **17** represent a rare class of organometallic species containing a nucleophilic selenolate moiety; notwithstanding, they can be readily purified by alumina chromatography (Figure 5) and handled in air for short times.

**Figure 5:**
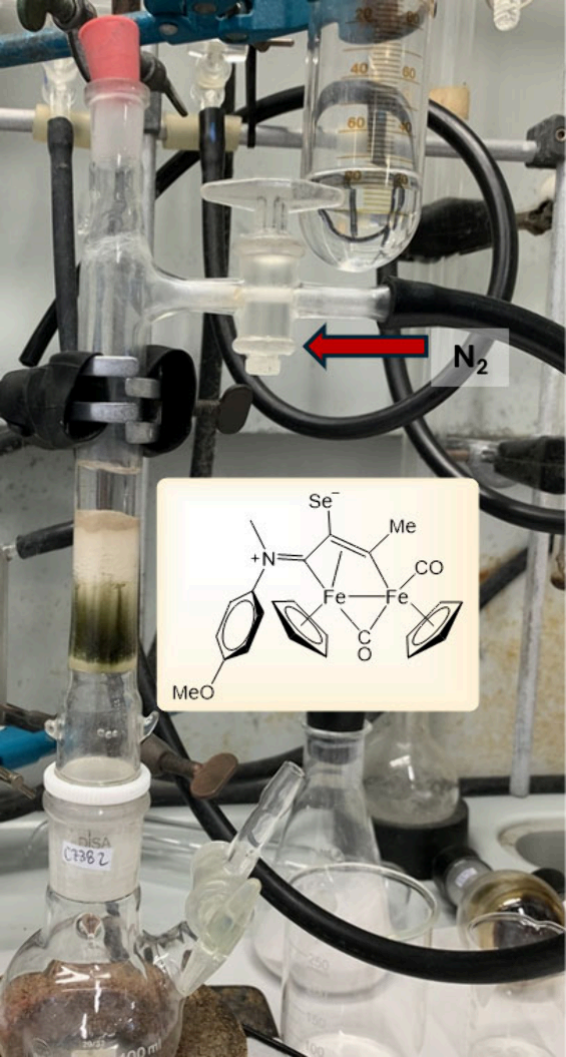
Alumina chromatography of a type **17** green complex.

**Scheme 11:**
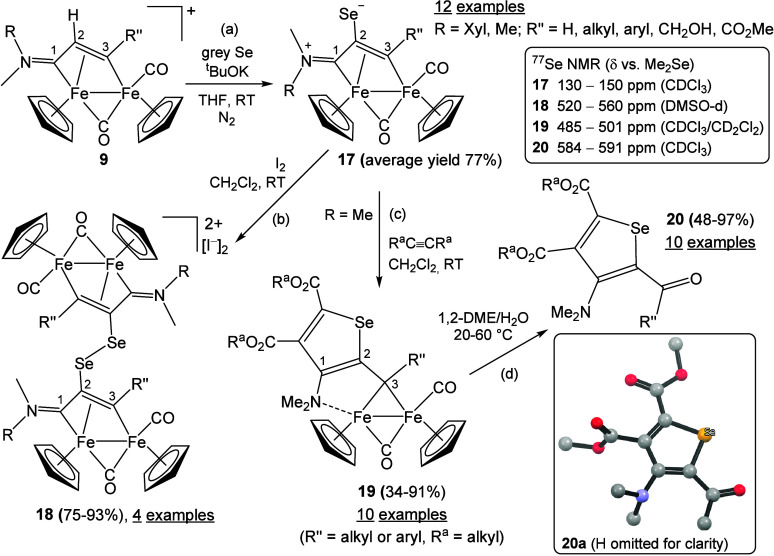
Synthesis of Selenolate–Vinyliminium Complexes (a), Iodine-Induced Oxidation to Fe_4_ Dimers (b), Alkyne Cyclization (c), and Isolation of Organic Selenophenes (d)^a^

a The insets show the ranges of ^77^Se NMR data across the series 17−20 and the molecular structure of a representative selenophene (**20a**).

Crystallographic analysis of one member (R = Xyl, R′ = *p*-tolyl) shows a C^2^–Se bond length of 1.899(3) Å. This value is comparable to the Csp_2_–Se distance in (tosyl)­(Ph)­C=C­(Ph)­(SeH), 1.905(2) Å, and longer than typical C=Se bonds in selenoketones (1.79–1.85 Å)^98,99^ and selenoamides (1.82–1.87 Å).^100,101^ The near-pure selenolate character of **17** renders these complexes highly reactive toward electrophiles; for example, even addition of CH_2_Cl^+^ occurs slowly in dichloromethane at room temperature.^102^ Oxidation with iodine rapidly induces dimerization of the diiron scaffold to give **18** (Scheme 11b):^102^ to our knowledge, this represents the only documented example of selenolate-to-diselenide dimerization involving a dimetallic framework. Dimerization of selenolate units in metal complexes is otherwise rare, having been observed in ferrocenes^103,104^ and cobaltocenium selenolate,^105^ and typically proceeds via *in situ* air oxidation of freshly prepared monomers.

The selenolate in **17** also reacts with internal alkynes bearing electron-withdrawing substituents, undergoing cyclization at C^1^ to give multisite-coordinated selenophene–alkylidene complexes **19** (Scheme 11c).^95,106^ The bridging alkylidene character of C^3^ is evidenced by a diagnostic ^13^C NMR resonance around 185 ppm.^32^ When we stored one member of **19** (R = Me, R′ = Et; ∼50 mg) as a powder in a sealed vial under air, we observed gas evolution upon opening after several months, and IR analysis of the residue confirmed loss of CO ligands. Optimization studies revealed that stirring 1,2-dimethoxyethane/H_2_O solutions of **19** under air promotes oxygen transfer and release of the Fischer alkylidene ligand (Scheme 11d). The resulting 2-acylselenophenes **20** are purified by alumina chromatography from cyclopentadiene and iron oxides. DFT and electrochemical studies suggest that both H_2_O and O_2_ can serve as sources of the acyl oxygen. Overall, the stepwise assembly of **20a** from Fp_2_ is summarized in Scheme 12. The unprecedented substituent pattern in **20** justifies the use of Fe_2_Cp_2_(CO)_4_ as a sacrificial template in this unusual metal-mediated synthesis of a family of compounds featuring a widely studied organic scaffold.^107^

**Scheme 12:**
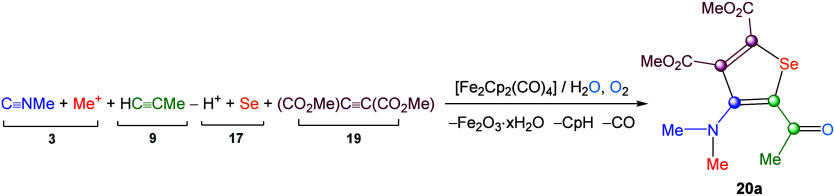
Stepwise Assembly of Molecular Building Blocks to Generate the Functionalized Selenophene Scaffold of **20a** from Fp_2_ via Diiron Complex Formation and Final μ-Alkylidene Release

### 3.4 Ferrabenzenes

Metallabenzenes represent an intensively investigated class of compounds that has fascinated chemists for decades owing to their unconventional aromaticity and potential for novel reactivity and applications.^108,109^ Most reported examples concern 4d and 5d late transition metals, particularly Ru, Os, Ir, and Pt, whereas systems based on 3d metals remain exceptionally rare. Notably, until 2025 the only ferrabenzene described in the literature was a η^6^-ferrabenzene–tungsten complex, obtained accidentally in low yield and lacking reproducibility.^110^ Recently, we unveiled the synthesis of the first family of ferrabenzenes.

The reactions of various vinyliminium complexes with ethyl diazoacetate, in the presence of a base, afford hydrazone-(bis)­alkylidene derivatives **21** (Scheme 13a).^111,76^ Complexes bearing R = Xyl can be isolated and are stable under an N_2_ atmosphere. By contrast, compounds bearing less sterically demanding R substituents are significantly less stable and can only be employed freshly for subsequent derivatization via N^1^-alkylation.^76,111^ In selected cases, a complex rearrangement occurs within a few hours in solution, leading to air-sensitive sandwich cyclohexadienone complexes **22** (Scheme 13b).^112^ The one-pot cyclization process involves coupling of two carbene units and one carbonyl ligand (C^1^ + C^5^ + C^4^), resulting in formation of two new C–C bonds. This transformation is sensitive to subtle steric and electronic factors, as clean formation of **22** has only been observed for complexes bearing R″ = Cy.

**Scheme 13:**
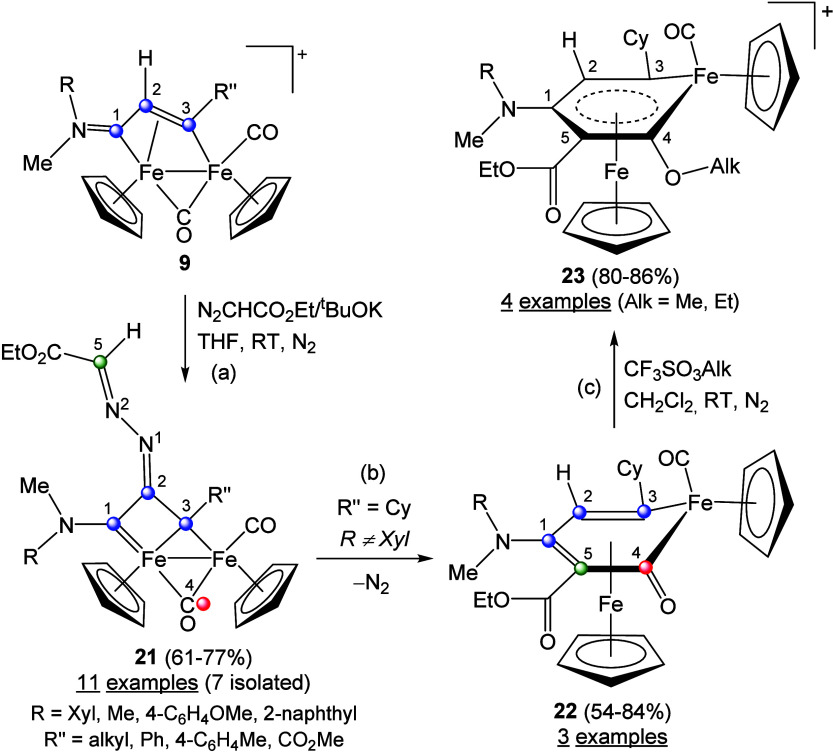
Vinyliminium Functionalization with Ethyl Diazoacetate (a) and Subsequent Intramolecular Rearrangement in Solution, Forming a Six-Membered Ferracycle (b), Followed by Alkylation Affording Iron-Coordinated Ferrabenzenes (c)

The mechanism of the **21** to **22** conversion has been investigated computationally. The initial step involves nucleophilic attack of the methyne carbon C^5^ onto the iminium carbon C^1^. Accordingly, the steric hindrance imposed by the *m*-xylyl substituent, which prevents nucleophilic approach to C^1^ (Scheme 8), accounts for the stability of the corresponding complexes **21**. Following C^5^–C^1^ bond formation, N_2_ extrusion occurs, and the bridging CO ligand becomes incorporated into the six-membered ring. NMR experiments employing either a C^2^D-labeled vinyliminium precursor or deuterated THF as solvent indicate that neither source contributes the final C^2^H proton in **22**. Excess ethyl diazoacetate is therefore proposed to act as a proton shuttle, facilitating intramolecular H-migration from C^5^ to C^2^. The air-stable iron-coordinated ferrabenzenes **23** are subsequently obtained via straightforward O-alkylation with alkyl triflates (Scheme 13c).

The metallaromatic character of the FeC_5_ ring in **23** is supported by crystallographic, NMR and computational analyses. The molecular structure of **23a** (Figure 6) displays C–C bond lengths in the 1.408(4)–1.436(4) Å range, indicative of extensive π-delocalization, along with C-centered bond angles of 121.1(3)–126.9(3)°, consistent with sp^2^ hybridization.

**Figure 6:**
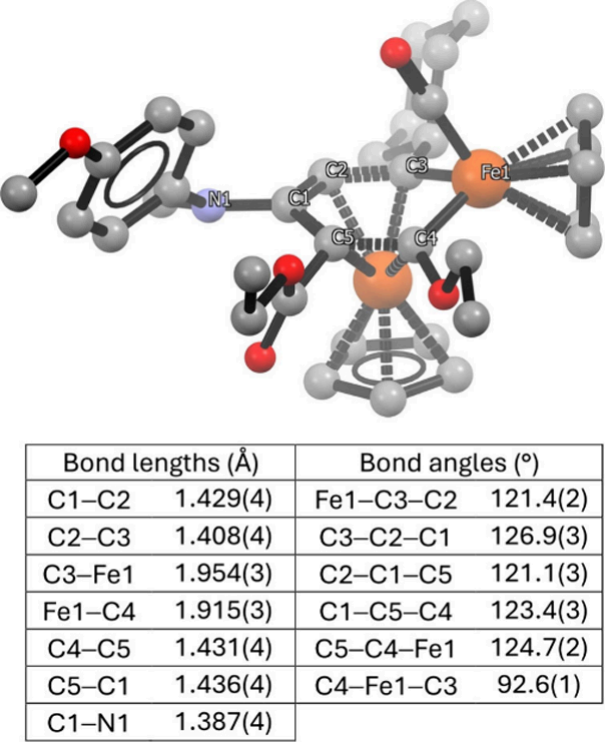
Molecular structure of representative iron-coordinated ferrabenzene **23a** (H atoms omitted).

In the ^1^H NMR spectra of **23a**, the C^2^H resonance appears at 6.40 ppm, closely matching that of [FeCp­(η^6^-benzene)]^+^ (6.50 ppm).^113^ In the ^13^C spectra, C^3^ and C^4^ exhibit downfield resonances at 221.9 and 235.6 ppm, respectively, reflecting their partial alkylidene (Fe=C) character.^32^ The chemical shifts of C^5^ (101.7 ppm), C^2^ (73.8 ppm) and C^1^ (130.2 ppm) further support aromatic delocalization. Notably, the C^1^ and C^2^ values fall within the expected ranges for dimethylamino-^114^ and alkoxo-substituted^115^ ring carbons in related metal-coordinated arenes.

The aromaticity of **23a** has further been assessed by calculating nucleus-independent chemical shift (NICS) values and the net ring current strength (*J* ^*ind*^) derived from MICD analyses. The resulting values (17.5 and 8.1, respectively) clearly indicate aromatic character. For comparison, the corresponding values calculated for 3-methoxy-*N,N*-dimethyl­aniline are 8.7 and 8.5, respectively.

## 4 Anticancer Studies

Organoiron complexes, particularly ferrocene derivatives, have attracted considerable attention as anticancer drug candidates.^116,117^ Their mechanism of action is generally attributed to intracellular Fe^II^-to-Fe^III^ oxidation, leading to increased levels of reactive oxygen species (ROS). Importantly, iron is an endogenous metal, a feature expected to limit the side effects of iron-based drugs.

In this context, diiron­(I) complexes of types **3** and **9**, together with several examples of type **5**, display key prerequisites for anticancer applications, representing a potential improvement over monoiron species:

1) Ready accessibility through straightforward and scalable synthetic protocols.
2) Permanent air stability and adequate aqueous solubility and stability, ensured by cationic nature and triflate counteranion. For instance, 70–88% of complexes **3** were recovered after incubation in DMEM cell culture medium for 72 h at 37 °C;^24^ under analogous conditions, complexes **9** can exhibit nearly quantitative stability.^74^
3) Wide structural variability, enabling fine-tuning of physicochemical and biological properties.
4) Balanced hydrophilic/lipo­philic character, with octanol/water partition coefficients (Log *P*_*ow*_) typically in the −1 to +1 range. While sufficient lipo­philicity is required for cellular uptake by passive diffusion, excessive lipo­philicity is unfavorable *in vivo*, contributing to systemic toxicity, with Log *P*_*ow*_ values >3 considered critical.^118^

On this basis, since 2018 we have screened the anticancer potential of the cationic derivatives described in this Account. A selection of the most promising compounds is shown in Scheme 14 together with representative biological data referenced to the clinical metallodrug cisplatin.

**Scheme 14:**
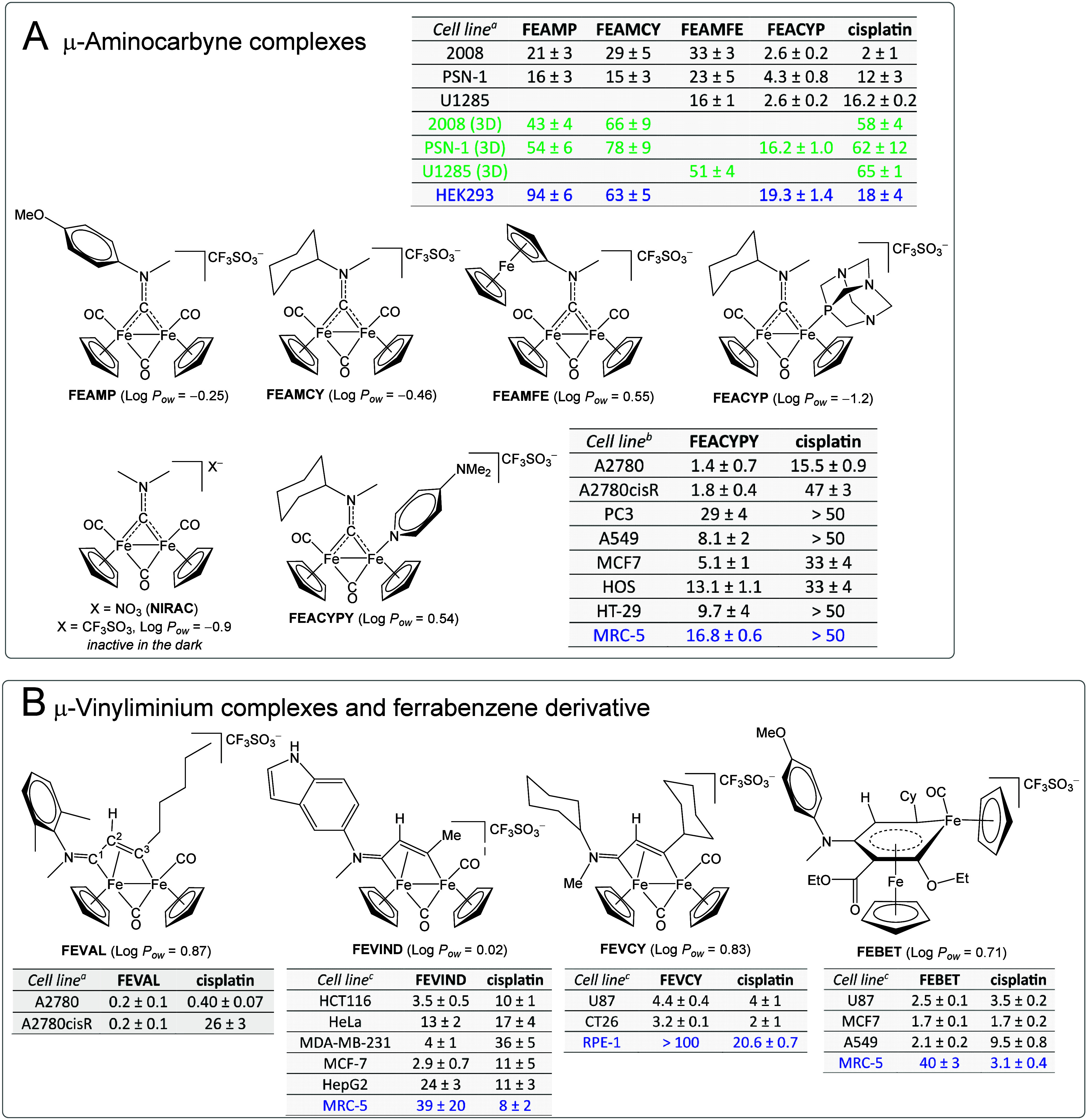
Selected Diiron­(I) Complexes Showing Promising Anticancer Activity, with Related Log *P*_*ow*_ Coefficients and IC_50_ Data (μM)^a^

a IC_50_ values from 3D cellular models are shown in green, while those from noncancerous cell lines are shown in blue. Incubation times (noted in the “Cell line” columns): (a) 72 h, (b) 24 h, and (c) 48 h.

Type **3** complexes display moderate *in vitro* cytotoxicity toward cancer cell lines, with preferential effects over noncancerous cells, associated with perturbation of cellular redox homeostasis (Scheme 14A).^24^ However, in three-dimensional cellular models, which are considered more predictive of *in vivo* efficacy, selected compounds retain activity more efficiently than cisplatin, achieving comparable or slightly superior performance.^24,39^ Fluorometric assays on FEAMP and FEAMCY revealed significant CO-releasing activity in PSN-1 cells,^119^ while markedly lower CO levels were detected in noncancerous HEK293 cells.^24^ These results suggest that antiproliferative effects correlate with intracellular disassembly of the diiron scaffold, a process that proceeds much more slowly in aqueous solutions.^120^ Consistently, NIRAC is inactive under standard assay conditions,^24,120^ but its cytotoxicity becomes appreciable upon photoinduced CO dissociation.^121^ The effect of CO monosubstitution was examined by evaluating the anticancer potential of several complexes of type **5**.^55,56,58^ In particular, FEACYPY and FEACYP show strong cytotoxicity combined with selectivity, as evidenced by reduced activity in noncancerous HEK293 and MRC-5 cells.^58,56^ FEAMP, FEAMCY, and FEACYP share the ability to target and inhibit thioredoxin reductase (TrxR), a cellular selenoenzyme playing a key role in redox regulation.^24,58^ Based on its outstanding profile, including remarkable aqueous solubility (∼15 g/L) and stability (90% intact after 24 h incubation in DMEM at 37 °C), FEACYP was investigated *in vivo* in a murine LLC carcinoma model. At a dose of 8 mg·kg^–1^, it produced excellent tumor growth inhibition (88% at day 15), without signs of systemic toxicity and with minimal body weight loss.^58^

Over 80 vinyliminium complexes (**9**) have been tested across several cancer cell lines under a variety of experimental conditions (Scheme 14B).^70–72,74–77^ Their activity depends on ligand substitution, roughly correlates with lipophilicity, and generally shows a tendency toward cancer cell selectivity. Mechanistic studies indicate a mode of action similar to **3** and **5**, mainly involving disruption of cellular redox balance. A chemometric analysis of IC_50_ data for 50 vinyliminium compounds against A2780 cells highlights the advantage of C^3^-alkyl over aryl substituents.^77^ In particular, a C^3^-cyclohexyl group (see FEVCY) imparts a beneficial effect on selectivity,^76^ whereas C^3^-carbohydrate functionalization (Scheme 6) is detrimental.^78^ FEVAL exemplifies an optimized design, combining moderate lipophilicity with nanomolar cytotoxicity against both A2780 and cisplatin-resistant A2780cisR cells,^77^ thus demonstrating the potential of vinyliminium complexes to overcome resistance mechanisms typical of platinum-based drugs.^122^ A family of N-indolyl complexes (e.g., FEVIND) exhibits a remarkable cytotoxicity profile.^74^ Mechanistic investigations reveal interactions with mitochondrial DNA and proteins, together with ROS-scavenging activity, ultimately leading to apoptosis. Interaction with nuclear DNA appears generally negligible for complexes **9**, unless tailored bioactive fragments are incorporated into the vinyliminium (e.g., chlorambucil, see Scheme 6).^79^ Finally, cationic ferrabenzene complexes retain the stability features of their synthetic precursors; the ethyl derivative FEBET stands out for its pronounced cytotoxicity and selectivity, associated with enhanced ROS generation (Scheme 14B).^112^

## 5 Concluding Remarks

Diiron bis­(cyclopentadienyl) complexes featuring bridging iminium ligands are readily accessible through broad-scope synthetic routes and enable a wide range of organometallic transformations, finely tuned by judicious choice of substituents. Functionalization of the vinyliminium ligand can proceed via multiple reaction pathways, opening avenues to complex organometallic architectures, heterometallic assemblies, ferracyclic derivatives, and unusually functionalized organic frameworks. The distinctive behavior arising from specific combinations of ligand sets and organic reagents, which governs stereochemical features and unlocks otherwise inaccessible reactivity patterns, suggests that Fe_2_Cp_2_(CO)_*x*_-based systems still hold substantial untapped potential. The recent discovery of the first family of ferrabenzenes exemplifies this capacity to uncover genuinely new organometallic motifs.

The “third age” of this chemistry has opened the door to applications, with readily accessible cationic complexes emerging as particularly attractive for medicinal purposes due to their favorable prerequisites for drug development. Promising results have already been obtained in 2D and 3D cellular models and, in selected cases, *in vivo*.^123^ The abundance of synthetic opportunities and structural diversity supports this direction, pointing to anticancer agents characterized by low systemic toxicity and optimized activity against specific tumor types. While significant efforts are currently underway, increasingly advanced biological investigations will be essential to fully realize this potential.

## Abbreviations

2008: human pancreatic cancer cell line
A2780: human ovarian cancer cell line
A2780cisR: cisplatin-resistant human ovarian cancer cell line
A375: human melanoma cell line
A431: human epidermoid carcinoma cell line
A549: human lung adenocarcinoma cell line
Cp: cyclopentadienyl
CT26: murine colon carcinoma cell line
Cy: cyclohexyl
DMEM: Dulbecco’s Modified Eagle Medium
Fc: ferrocenyl
Fp_2_: diiron bis­(cyclopentadienyl) tetracarbonyl
HBPin: pinacolborane
HCT-15: human colorectal adenocarcinoma cell line
HCT116: human colorectal carcinoma cell line
HeLa: human cervical cancer cell line
HEK293: human embryonic kidney cell line (noncancerous)
HepG2: human hepatocellular carcinoma cell line
HOS: human osteosarcoma cell line
IC_50_: half-maximal inhibitory concentration
IR: infrared spectroscopy
LLC: murine Lewis lung carcinoma
MCF-7: human breast adenocarcinoma cell line
MCF-7-ADR: doxorubicin-resistant human breast cancer cell line
MDA-MB-231: human triple-negative breast cancer cell line
Me: methyl
MICD: gauge-including magnetically induced current density
MRC-5: human lung fibroblast cell line (noncancerous)
NICS: nucleus-independent chemical shift
NMR: nuclear magnetic resonance
PC3: human prostate cancer cell line
Ph: phenyl
PSN-1: human pancreatic cancer cell line
PTA: 1,3,5-triaza-7-phosphaadamantane
RPE-1: human retinal pigment epithelial cell line (noncancerous)
ROS: reactive oxygen species
TrxR: thioredoxin reductase
U1285: human small-cell lung cancer cell line
U2185: human glioblastoma cell line
U87: human glioblastoma cell line
X-ray: X-ray crystallography
Xyl: *meta*-xylyl
tosyl: *para*-toluenesulfonyl
